# Administrative regulation-informed analysis of the developmental path of national volume-based procurement to improve drug accessibility in China

**DOI:** 10.3389/fpubh.2024.1342632

**Published:** 2024-07-10

**Authors:** Songxin Lu, Xiangdong Liu, Zhengzong Huang, Zhiheng Zhou, Zehua Feng

**Affiliations:** ^1^School of Economic, Shenzhen Polytechnic University, Shenzhen, China; ^2^Faculty of Humanities and Social Sciences, Macao Polytechnic University, Macao, China; ^3^College of Humanities and Social Sciences, Shenzhen Technology University, Shenzhen, China; ^4^Pingshan Hospital, Southern Medical University, Shenzhen, China; ^5^School of Law, Guangdong University of Technology, Guangzhou, China

**Keywords:** national volume-based procurement, evolutionary game, drug accessibility, administrative regulation, generic drug

## Abstract

**Introduction:**

The procurement of medicines via China’s national volume-based procurement (NVBP) necessitates collaboration among various entities. This paper highlights the legal significance of the engagement of pharmaceutical companies, hospitals, and the National Healthcare Security Administration (NHSA) in improving drug accessibility.

**Methods:**

We conducted a numerical simulation using MATLAB to develop an evolutionary game model involving these three participants in NVBP.

**Results:**

Our findings indicate that the final evolutionary stabilization strategies are pharmaceutical companies actively participating, hospitals using bid-winning medicines, and the NHSA implementing a low-intensity intervention. The study reveals that the evolutionary outcomes for hospitals and pharmaceutical companies are significantly affected by factors such as NHSA’s subsidy level and pharmaceutical companies’ level of participation. However, NHSA’s decision-making process is less influenced by these factors.

**Discussion:**

From a legal perspective, the successful implementation of NVBP, ensuring fairness and legality, requires adherence to relevant policies and regulations. The NHSA should employ statutory incentives and regulatory methods in formulating and adjusting NVBP policy to enable pharmaceutical companies, hospitals, and the NHSA to exercise their rights rationally within the legal framework of the game process.

## 1 Introduction

China’s central government formally enforced a national volume-based procurement (NVBP) policy on 1 January 2019. By consolidating national purchasing power and exchanging large volumes for reduced drug costs, the policy aims to decrease market prices and increase drug accessibility (1). The introduction of a substantial quantity of bid-winning generic drugs into hospitals, concurrent with the implementation of China’s pilot NVBP system, has led to a steep decline in drug prices and necessitated significant adjustments in the generic drug production market (2). Bid-winning drugs refer to those generic drugs that have won bids under the NVBP policy, allowing them to be purchased in large volumes at reduced prices. A detailed list of these medicines is published by the Joint Procurement Office for Medicines of State Organizations of China (3). As an alternative to expensive pharmaceuticals, generic drugs are the primary option in most developing nations. In manufacturing generics, effectiveness and cost must coexist. Despite their heavy use in Chinese hospitals, generics are not necessarily inexpensive (4). Access to medicines in China has been restricted due to the slow materialization of the “patent cliff” phenomenon (5). Therefore, employing administrative regulation to reduce medication costs and enhance accessibility is essential.

Drug procurement is a critical component of the healthcare system, directly impacting drug quality, pricing, and patient safety. In China, public medical institutions primarily conduct drug procurement through centralized platforms at the provincial or municipal level. These platforms ensure transparency and market competition through public and competitive bidding, thereby guaranteeing the quality and reasonable pricing of drugs. In recent years, primary medical institutions, which form the foundation of the medical service system, have actively participated in state-organized centralized drug procurement projects. Notably, programs like the “4 + 7” volume-based purchasing program have allowed primary healthcare institutions to acquire high-quality medications at reduced prices, significantly alleviating patients’ financial burdens. Through centralized procurement and volume-for-price exchanges, these programs have led to a substantial decrease in drug prices, offering patients more affordable medication options. The active involvement of primary care institutions not only enhances the efficiency and transparency of drug procurement but also promotes the optimal allocation of medical resources. Consequently, this participation has provided tangible benefits to patients, enabling them to access higher quality medical services at lower costs.

This research aims to analyze the influence and interaction of three key factors on the availability of medicines in China: pharmaceutical companies, hospitals, and the National Healthcare Security Administration (NHSA). Specifically, we seek to address the following research questions and hypotheses: How do pharmaceutical companies adjust their strategies in response to NVBP policies? We hypothesize that pharmaceutical companies will lower drug prices and increase production capacities to meet NVBP requirements. What role do hospitals play in the NVBP system, and how do their procurement practices influence drug availability? We hypothesize that hospitals’ active participation in centralized procurement will lead to more efficient drug distribution and improved patient access to medications. How does NHSA’s administrative regulation impact the dynamics between pharmaceutical companies and hospitals? We hypothesize that NHSA’s regulatory measures will balance economic efficiency and drug accessibility, fostering collaboration among stakeholders. By investigating these questions, we aim to provide a comprehensive understanding of the NVBP system’s operational dynamics and offer insights into optimizing the availability and affordability of medicines in China.

In China, payment for drug costs primarily depends on medical insurance and patients’ out-of-pocket expenses. Medical insurance, a vital component of the social security system, provides partial or full payment coverage for drug costs in most public hospitals. Patients with different types of medical insurance, such as urban workers’ medical insurance and urban and rural residents’ medical insurance, receive varying levels of reimbursement for drug costs. The specific reimbursement rate and scope are typically determined by the health insurance policy and the patient’s insurance type. The diversity of health insurance policies aims to reduce patients’ financial burden and improve access to healthcare services. However, not all medications are covered by health insurance. Patients often have to pay out-of-pocket for drugs not included in Medicare or those outside Medicare coverage, including high-priced prescription drugs with special efficacy or new drugs not yet listed in the health insurance catalog. These expenses can impose financial pressure on patients, who must pay based on their financial capacity. To alleviate this financial burden, the government and community must collaborate to continuously improve health insurance policies, expand the health insurance catalog, increase reimbursement rates, and regulate drug prices to ensure they are reasonable and fair. Such measures will better meet patients’ medication needs and enhance the quality of medical services.

Drug costs and medical practice engagement are crucial issues directly impacting the financial burden on patients and the sustainability of healthcare services. High drug prices place a significant burden on patients, potentially leading them to delay or forgo necessary treatments due to financial pressure. This not only exacerbates the financial hardships of patients and their families but also negatively affects the overall health of the community. To address the issue of high drug prices, various measures have been adopted by medical practice participants to safeguard patients’ interests. Medical practices are strengthening cooperation with drug suppliers and reducing drug procurement costs through centralized purchasing and volume–price negotiations, thereby alleviating the medication burden on patients. Additionally, policy efforts are actively addressing high drug prices. The government is lowering medication costs for patients through drug pricing policies, the implementation of health insurance systems, and other measures. Furthermore, the government encourages medical institutions and drug manufacturers to engage in technological innovation and research and development to introduce more effective and reasonably priced drugs to meet patients’ needs. Addressing drug costs and medical practice engagement requires the collective attention of society. Only through the efforts of multiple parties can drug costs be effectively reduced, patients’ interests protected, and the quality and sustainability of medical services improved.

Pharmaceutical companies participate in NVBP primarily to enhance drug sales volume and revenue and to stabilize market expectations. This participation aims to optimize economic benefits. When pharmaceutical companies win a bid, Chinese public hospitals are obligated to make prompt payments in accordance with the procurement agreement. The National Healthcare Security Administration’s (NHSA) medical insurance fund must also provide public hospitals with a minimum advance of 30 percent of the procurement amount. Drug prices are a significant indicator of NVBP (6). Pharmaceutical companies must reduce their product costs to improve their bidding prospects. Moreover, to meet NHSA’s centralized purchasing volume requirement, they must acquire additional production facilities and equipment to increase manufacturing capabilities. The short-term economic efficiency of drug companies will be impacted by factors such as decreased drug prices and increased production expenses. In light of this, the NHSA must facilitate administrative incentives for NVBP to motivate pharmaceutical companies. For example, NHSA should ensure that these companies have adequate financial resources to expand production capabilities and guarantee timely deliveries.

High drug prices can prevent many patients from receiving timely treatment, endangering public health and potentially undermining the government’s credibility. Pharmaceutical companies, driven by financial gains, often provide kickbacks to medical facilities that utilize expensive medications, engaging in commercial bribery (7). These “sales costs” are ultimately funded by patients and health insurance funds. The propensity of hospitals to use certain pharmaceuticals significantly influences the distribution and sale of drugs. Despite the abundance of public hospitals in China, medical resources remain scarce relative to the substantial population. To improve the standard of treatment and ensure operational continuity, many public hospitals have adopted the practice of “supporting hospitals with drugs.” They generate revenue by selling expensive drugs to patients, using additional funds to cover operating expenses and enhance the welfare of their medical staff.

Currently, scholarly investigations have thoroughly examined the development process and operational mode of NVBP in China. These studies emphasize the importance of involving a diverse range of stakeholder groups in common governance. However, there is a lack of discussion on how the principal actors collaborate to guide the pharmaceutical market toward increased drug accessibility and productivity, especially when viewed as a dynamic game process. Most research on NVBP focuses on data analysis (8, 9), empirical cases (10, 11), policy design (1), and implementation effects (12, 13). Although some researchers have analyzed commercial competition among pharmaceutical companies using the event study approach (10), few have examined the strategic interaction between pharmaceutical companies, hospitals, and the NHSA. As primary consumers of medications, hospitals significantly influence NHSA’s purchasing decisions. Actions taken by pharmaceutical companies and the NHSA aim to benefit hospitals. Therefore, this research includes hospitals as game subjects, establishes a three-party evolutionary game model, and investigates the most efficient operational mode for the NVBP system under administrative regulation. It also analyzes the factors each party should consider.

## 2 Methods

### 2.1 Basic assumptions

*Hypothesis 1*: The implementation of NVBP necessitates the involvement of pharmaceutical companies, public hospitals, and the NHSA. This paper focuses on these three participants.

*Hypothesis 2*: The action strategies of the participants are as follows: pharmaceutical companies can adopt either positive or negative participation in NVBP; public hospitals can use either bid-winning or non-bid-winning drugs in NVBP; and NHSA can implement either high-intensity or low-intensity intervention in NVBP.

*Hypothesis 3*: Each participant possesses finite rationality. This assumption is grounded in the behavioral economic theory which posits that individuals and organizations operate under bounded rationality due to limitations in information and cognitive processing capabilities (14). Due to information asymmetry and the premise that game participants wish to maximize expected benefits, they are unable to select the optimal strategy for a single game.

*Hypothesis 4*: Pharmaceutical companies that opt for the “negative participation in NVBP” strategy retain the ability to bribe public hospitals and profit from the sale of non-bid-winning drugs. Subsequently, NHSA should levy penalties on pharmaceutical companies in an effort to encourage their accountability in the realms of drug price reduction and accessibility enhancement.

*Hypothesis 5*: This paper operates under the assumption that the overall benefit derived by public hospitals can be categorized into the following components: the economic benefit obtained from utilizing the bid-winning drugs, denoted as *R_3_*; the economic benefit from utilizing the non-bid-winning drugs, denoted as *R_4_*; the subsidy provided by the NHSA, denoted as *S_2_;* the cost of using the bid-winning drugs, denoted as *C_4_*; and the cost of using the non-bid-winning drugs, denoted as *C_5_*. The benefits of positive participation in NVBP by pharmaceutical companies are denoted as *R_1_* and the associated costs as *C_2_.* The benefits of negative participation in NVBP by pharmaceutical companies are denoted as *R_2_* and the costs as *C_3_*. *S_1_* denotes the subsidies given to pharmaceutical companies by NHSA. *S_2_* also refers to the budget of the health insurance fund given to public hospitals by the NHSA. *S_3_* denotes the additional subsidies provided to hospitals by the NHSA to promote the NVBP when pharmaceutical companies adopt negative participation. *K* denotes the fine imposed by the NHSA on pharmaceutical companies for commercial bribery. *T* represents the commercial bribe given by pharmaceutical companies to public hospitals. *P* denotes the benefits accrued to the pharmaceutical companies from the public hospitals’ use of bid-winning drugs. The NHSA incurs regulatory costs, denoted as *C_1_*, to maintain the orderly operation of the NVBP. *V* represents the loss to NHSA’s reputation, with *V* being greater than *C_1_*. *θ* denotes the proportion of bid-winning drug prices that decrease when pharmaceutical companies participate in the NVBP. *ε* represents the proportional decline in revenue for pharmaceutical companies from the use of bid-winning drugs by public hospitals when these companies participate negatively in NVBP. *G_1_* denotes the social benefits to NHSA from the positive participation of pharmaceutical companies in NVBP. *G_2_* denotes the social benefits to the NHSA from the hospitals’ use of bid-winning drugs.

As shown in Table 1, we derived the payment matrix for the “NHSA-pharmaceutical companies–public hospitals” tripartite evolution game based on the aforementioned assumptions and various decisions.

**Table 1:** Payment matrix.

		Hospital use of bid-winning drugs y			Hospitals use non-bid-winning drugs(1−y)		
		Pharmaceutical company	Public hospital	NHSA	Pharmaceutical company	Public hospital	NHSA
High-intensity intervention by NHSA ( z )	Pharmaceutical companies positively participate in NVBP ( x )	$R_{1}-C_{2}+S_{1}+P$	$R_{3}+S_{2}-\theta C_{4}$	$G_{1}+G_{2}-C_{1}-S_{1}-S_{2}$	$R_{1}-C_{2}+S_{1}$	$R_{4}-C_{5}$	$G_{1}-C_{1}-S_{1}$
	Pharmaceutical companies negatively participate in NVBP ( 1−x )	$R_{2}-C_{3}+\varepsilon P$	$R_{3}+S_{2}-C_{4}+S_{3}$	$G_{2}-C_{1}-S_{2}$	$R_{2}-C_{3}-K-T$	$R_{4}-C_{5}+T$	$K-C_{1}$
Low-intensity intervention by NHSA ( 1−z )	Pharmaceutical companies positively participate in NVBP ( x )	$R_{1}-C_{2}+S_{1}+P$	$R_{3}+S_{2}-\theta C_{4}$	$G_{1}+G_{2}-S_{1}-S_{2}$	$R_{1}-C_{2}+S_{1}$	$R_{4}-C_{5}$	$G_{1}-S_{1}$
	Pharmaceutical companies negatively participate in NVBP ( 1−x )	$R_{2}-C_{3}+\varepsilon P$	$R_{3}+S_{2}-C_{4}$	$G_{2}-S_{2}-V$	$R_{2}-C_{3}-T$	$R_{4}-C_{5}+T$	−V

### 2.2 Model analysis

The expected benefits, as well as the average expected benefits ( $U_{11}$ , $U_{12}$ , ${\bar{U}}_{1}$ ), for positive or negative participation in NVBP by pharmaceutical companies are as follows:


$$\begin{array}{l} U_{11}=yz\left(R_{1}-C_{2}+S_{1}+P\right)+y\left(1-z\right)\left(R_{1}-C_{2}+S_{1}+P\right)\\ +z\left(1-y\right)\left(R_{1}-C_{2}+S_{1}\right)+\left(1-y\right)\left(1-z\right)\left(R_{1}-C_{2}+S_{1}\right)=yP \end{array}$$

$$\begin{array}{l} U_{12}=yz\left(R_{2}-C_{3}+\varepsilon P\right)+y\left(1-z\right)\left(R_{2}-C_{3}+\varepsilon P\right)\\ +z\left(1-y\right)\left(R_{2}-C_{3}-T-K\right)+\left(1-y\right)\left(1-z\right)\left(R_{2}-C_{3}-T\right)\\ =yT-y\varepsilon P-zK+yzK+R_{2}-C_{3}-T \end{array}$$

$${\bar{U}}_{1}=xU_{11}+\left(1-x\right)U_{12}$$

The replication dynamic equation for the pharmaceutical company strategy is as follows:


$$\begin{array}{l} F\left(x\right)=\frac{dx}{dt}=x\left(U_{11}-{\bar{U}}_{1}\right)=x\left(1-x\right)\left(U_{11}-U_{12}\right)\\ =x\left(1-x\right)\left(yP-yT+y\varepsilon P+zK-yzK-R_{2}+C_{3}+T\right) \end{array}$$

The public hospitals’ expected benefits from the use of bid-winning and non-bid-winning drugs, as well as the average expected benefits ( $U_{21}$ , $U_{22}$ , ${\bar{U}}_{2}$ ) are as follows:


$$\begin{array}{l} U_{21}=xz\left(R_{3}+S_{2}-\theta C_{4}\right)+x\left(1-z\right)\left(R_{3}+S_{2}-\theta C_{4}\right)\\ +z\left(1-x\right)\left(R_{3}+S_{2}-C_{4}+S_{3}\right)+\left(1-x\right)\left(1-z\right)\left(R_{3}+S_{2}-C_{4}\right)\\ =xC_{4}-x\theta C_{4}+zS_{3}-xzS_{3}+R_{3}+S_{2}-C_{4} \end{array}$$

$$\begin{array}{l} U_{22}=xz\left(R_{4}-C_{5}\right)+x\left(1-z\right)\left(R_{4}-C_{5}\right)+z\left(1-x\right)\left(R_{4}-C_{5}+T\right)\\ +\left(1-x\right)\left(1-z\right)\left(R_{4}-C_{5}+T\right)=R_{4}-C_{5}+T-xT \end{array}$$

$${\bar{U}}_{2}=yU_{21}+\left(1-y\right)U_{22}$$

The replication dynamic equation for the public hospital strategy is as follows:


$$\begin{array}{l} F\left(y\right)=\frac{\mathrm{dy}}{dt}=y\left(U_{21}-{\bar{U}}_{2}\right)=y\left(1-y\right)\left(U_{21}-U_{22}\right)\\ =y\left(1-y\right)\left(xC_{4}-x\theta C_{4}+zS_{3}-xzS_{3}+R_{3}+S_{2}-C_{4}-R_{4}+C_{5}-T+xT\right) \end{array}$$

The expected returns and the average expected returns ( $U_{31}$ , $U_{32}$ , ${\bar{U}}_{3}$ ) for strict or lax regulation by the NHS are as follows:


$$\begin{array}{l} U_{31}=xy\left(G_{1}+G_{2}-C_{1}-S_{1}-S_{2}\right)+x\left(1-y\right)\left(G_{1}-C_{1}-S_{1}\right)\\ +y\left(1-x\right)\left(G_{2}-C_{1}-S_{2}\right)+\left(1-x\right)\left(1-y\right)\left(K-C_{1}\right)\\ =xG_{1}-xS_{1}+yG_{2}-yS_{2}+K-C_{1}-xK-yK+xyK \end{array}$$

$$\begin{array}{l} U_{32}=xy\left(G_{1}+G_{2}-S_{1}-S_{2}\right)+x\left(1-y\right)\left(G_{1}-S_{1}\right)\\ +y\left(1-x\right)\left(G_{2}-S_{2}-V\right)+\left(1-x\right)\left(1-y\right)\left(-V\right)\\ =xG_{1}-xS_{1}+yG_{2}-yS_{2}-V+xV \end{array}$$

$${\bar{U}}_{3}=zU_{31}+\left(1-z\right)U_{32}$$

The replication dynamic equation for the NHSA strategy is as follows:


$$\begin{array}{l} F\left(z\right)=\frac{dz}{dt}=z\left(U_{31}-{\bar{U}}_{3}\right)=z\left(1-z\right)\left(U_{31}-U_{32}\right)\\ =z\left(1-z\right)\left(K-C_{1}-xK-yK+xyK+V-xV\right) \end{array}$$

The dynamic competition among pharmaceutical companies, public hospitals, and the NHSA is such that the success likelihood of any strategy changes over time. The stability of differential equations in a dynamic system dictates that all dynamics tend toward stability when the solutions to all dynamic equations equal zero. The calculation of the equilibrium points in this three-party evolutionary game is defined by the equations *F(x) = 0, F(y) = 0,* and *F(z) = 0*. This implies that:


$$F\left(x\right)=x\left(1-x\right)\left(yP-yT+y\varepsilon P+zK-yzK-R_{2}+C_{3}+T\right)=0$$

$$F\left(y\right)=y\left(1-y\right)\left(\begin{array}{l} xC_{4}-x\theta C_{4}+zS_{3}-xzS_{3}+\\ R_{3}+S_{2}-C_{4}-R_{4}+C_{5}-T+xT \end{array}\right)=0$$

$$F\left(z\right)=z\left(1-z\right)\left(K-C_{1}-xK-yK+xyK+V-xV\right)=0$$

There exist eight special equilibrium points: $E_{1}\left(0,0,0\right)$ , $E_{2}\left(1,0,0\right),\;E_{3}\left(0,1,0\right),E_{4}\left(0,0,1\right),E_{5}\left(1,1,0\right),E_{6}\left(1,0,1\right),E_{7}\left(0,1,1\right),E_{8}\left(1,1,1\right)$ . All the participants adopt a pure strategy at each equilibrium point. Furthermore, the stability of the equilibrium points in a differential system can be determined by analyzing the eigenvalues of the system’s Jacobian matrix, following Friedman’s method (15). The Jacobian matrix for this tripartite evolutionary game system is as follows:


$$J=\left[\begin{array}{ccc} \frac{\partial F\left(x\right)}{\partial x} & \frac{\partial F\left(x\right)}{\partial y} & \frac{\partial F\left(x\right)}{\partial z}\\ \frac{\partial F\left(y\right)}{\partial x} & \frac{\partial F\left(y\right)}{\partial y} & \frac{\partial F\left(y\right)}{\partial z}\\ \frac{\partial F\left(z\right)}{\partial x} & \frac{\partial F\left(z\right)}{\partial y} & \frac{\partial F\left(z\right)}{\partial z} \end{array}\right]=\left[\begin{array}{ccc} J_{11} & J_{12} & J_{13}\\ J_{21} & J_{22} & J_{23}\\ J_{31} & J_{32} & J_{33} \end{array}\right]$$

Among them:


$$J_{11}=\left(1-2x\right)\left(yP-yT+y\varepsilon P+zK-yzK-R_{2}+C_{3}+T\right)$$

$$J_{12}=x\left(1-x\right)\left(P_{1}-T+\varepsilon P-zK\right)$$

$$J_{13}=x\left(1-x\right)\left(K-yK\right)$$

$$J_{21}=y\left(1-y\right)\left(C_{4}-\theta C_{4}-zS_{3}+T\right)$$

$$J_{22}=\left(1-2y\right)\left(\begin{array}{l} xC_{4}-x\theta C_{4}+zS_{3}-xzS_{3}+\\ R_{3}+S_{2}-C_{4}-R_{4}+C_{5}-T+xT \end{array}\right)$$

$$J_{23}=y\left(1-y\right)\left(S_{3}-xS_{3}\right)$$

$$J_{31}=z\left(1-z\right)\left(-K+yK-V\right)$$

$$J_{32}=z\left(1-z\right)\left(-K+xK\right)$$

$$J_{33}=\left(1-2z\right)\left(K-C_{1}-xK-yK+xyK+V-xV\right)$$

An equilibrium point is deemed to be the system’s evolutionary stable strategy (ESS) when all of its eigenvalues are less than zero. Conversely, an equilibrium point is considered unstable if at least one of its eigenvalues is greater than zero.

For the equilibrium point $E_{1}\left(0,0,0\right)$ , the Jacobian matrix $\left(M_{1}\right)$ is as follows:


$$M_{1}=\left(\begin{array}{ccc} K-C_{1}+V & 0 & 0\\ 0 & C_{3}-R_{2}+T & 0\\ 0 & 0 & C_{5}-C_{4}+R_{3}-R_{4}+S_{2}-T \end{array}\right)$$

For the equilibrium point $E_{2}\left(1,0,0\right)$ , the Jacobian matrix ( $M_{2})$ is as follows:


$$M_{2}=\left(\begin{array}{ccc} -C_{1} & 0 & 0\\ 0 & R_{2}-C_{3}-T & 0\\ 0 & 0 & C_{5}+R_{3}-R_{4}+S_{2}-\theta C_{4} \end{array}\right)$$

For the equilibrium point $E_{3}\left(0,1,0\right)$ , the Jacobian matrix ( $M_{3}$ ) is as follows:


$$M_{3}=\left(\begin{array}{ccc} V-C_{1} & 0 & 0\\ 0 & C_{3}+P_{1}-R_{2}+\varepsilon P & 0\\ 0 & 0 & C_{4}-C_{5}-R_{3}+R_{4}-S_{2}+T \end{array}\right)$$

For the equilibrium point $E_{4}\left(0,0,1\right)$ , the Jacobian matrix ( $M_{4})$ is as follows:


$$M_{4}=\left(\begin{array}{ccc} C_{1}-K-V & 0 & 0\\ 0 & C_{3}+K-R_{2}+T & 0\\ 0 & 0 & C_{5}-C_{4}+R_{3}-R_{4}+S_{2}+S_{3}-T \end{array}\right)$$

For the equilibrium point $E_{5}\left(1,1,0\right)$ , the Jacobian matrix $\left(M_{5}\right)$ is as follows:


$$M_{5}=\left(\begin{array}{ccc} -C_{1} & 0 & 0\\ 0 & R_{2}-P_{1}-C_{3}-\varepsilon P & 0\\ 0 & 0 & R_{4}-R_{3}-C_{5}-S_{2}+\theta C_{4} \end{array}\right)$$

For the equilibrium point $E_{6}\left(1,0,1\right)$ , the Jacobian matrix $\left(M_{6}\right)$ is as follows:


$$M_{6}=\left(\begin{array}{ccc} C_{1} & 0 & 0\\ 0 & R_{2}-K-C_{3}-T & 0\\ 0 & 0 & C_{5}+R_{3}-R_{4}+S_{2}-\theta C_{4} \end{array}\right)$$

For the equilibrium point $E_{7}\left(0,1,1\right)$ , the Jacobian matrix $\left(M_{7}\right)$ is as follows:


$$M_{7}=\left(\begin{array}{ccc} C_{1}-V & 0 & 0\\ 0 & C_{3}+P_{1}-R_{2}+\varepsilon P & 0\\ 0 & 0 & C_{4}-C_{5}-R_{3}+R_{4}-S_{2}-S_{3}+T \end{array}\right)$$

For the equilibrium point $E_{8}\left(1,1,1\right)$ , the Jacobian matrix ( $M_{8})$ is as follows:


$$M_{8}=\left(\begin{array}{ccc} C_{1} & 0 & 0\\ 0 & R_{2}-P_{1}-C_{3}-\varepsilon P & 0\\ 0 & 0 & R_{4}-R_{3}-C_{5}-S_{2}+\theta C_{4} \end{array}\right)$$

The equilibrium points $E_{1}\left(0,0,0\right)$ , $E_{3}\left(0,1,0\right)$ , $E_{6}\left(1,0,1\right)$ , and $E_{8}\left(1,1,1\right)$ do not meet the necessary criteria for asymptotic stability in the evolutionary game system, as indicated by the computational analysis. As a result, these points cannot be classified as stable equilibrium points. Table 2 illustrates the evolutionary stability conditions corresponding to the other equilibrium points.

**Table 2:** Evolutionary stabilization conditions for the equilibrium points.

Equilibrium point	Evolutionary stabilization conditions
$E_{2}\left(1,0,0\right)$	$R_{2}-C_{3}-T<0$
	$C_{5}+R_{3}-R_{4}+S_{2}-\theta C_{4}<0$
$E_{4}\left(0,0,1\right)$	$C_{1}-K-V<0$
	$C_{3}+K-R_{2}+T<0$
	$C_{5}-C_{4}+R_{3}-R_{4}+S_{2}+S_{3}-T<0$
$E_{5}\left(1,1,0\right)$	$R_{2}-P-C_{3}-\varepsilon P<0$
	$R_{4}-R_{3}-C_{5}-S_{2}+\theta C_{4}<0$
$E_{7}\left(0,1,1\right)$	$C_{1}-V<0$
	$C_{3}+P_{1}-R_{2}+\varepsilon P<0$
	$C_{4}-C_{5}-R_{3}+R_{4}-S_{2}-S_{3}+T<0$

## 3 Results

This study derives three evolutionary stabilization strategies through simulation and calculation analysis. For a clearer observation of the evolutionary trajectory of the game’s participants and their sensitivity to parameters, we used MATLAB 2021a to simulate these evolutionary stabilization strategies and their associated parameters.

### 3.1 Evolutionary stabilization strategy

**Figure 1:**
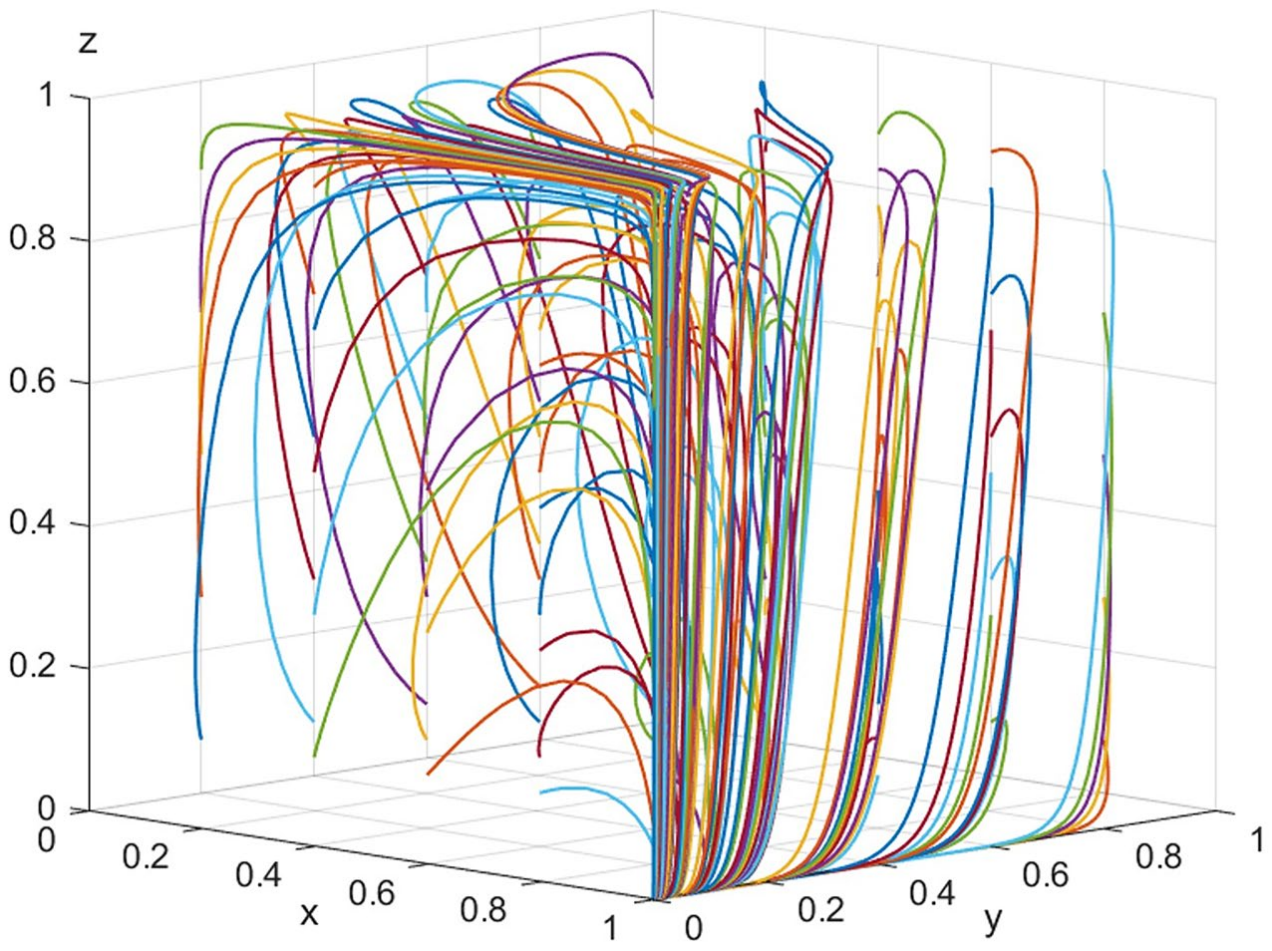
Simulation of the equilibrium point (1,0,0) parameters.

The equilibrium point *E_2_ (1,0,0)* is evolutionarily stable when the conditions *R_2_ − C_3_ − T < 0 and C_5_ + R_3_ − R_4_ + S_2_ − θC_4_ < 0* are met. To fulfill these conditions, assume the following values: *p = 100, T = 200, K = 100, R_2_ = 200, C_3_ = 100, C_4_ = 50, S_3_ = 40, R_3_ = 40, S_2_ = 40, R4 = 120, C_5_ = 50, C1 = 100, V = 500, ε = 0.5, θ = 0.5.* The final evolution result is (1,0,0), regardless of the hospital’s initial willingness to engage, as demonstrated in Figure 1. This outcome primarily stems from the fact that the benefits pharmaceutical companies gain from not participating in NVBP are less than zero. Additionally, the benefit hospitals receive from using bid-winning drugs is lower than that from using non-bid-winning drugs. Therefore, the optimal strategies for pharmaceutical companies, public hospitals, and NHSA are “positive participation,” “use of non-bid-winning drugs,” and “low-intensity intervention,” respectively.

**Figure 2:**
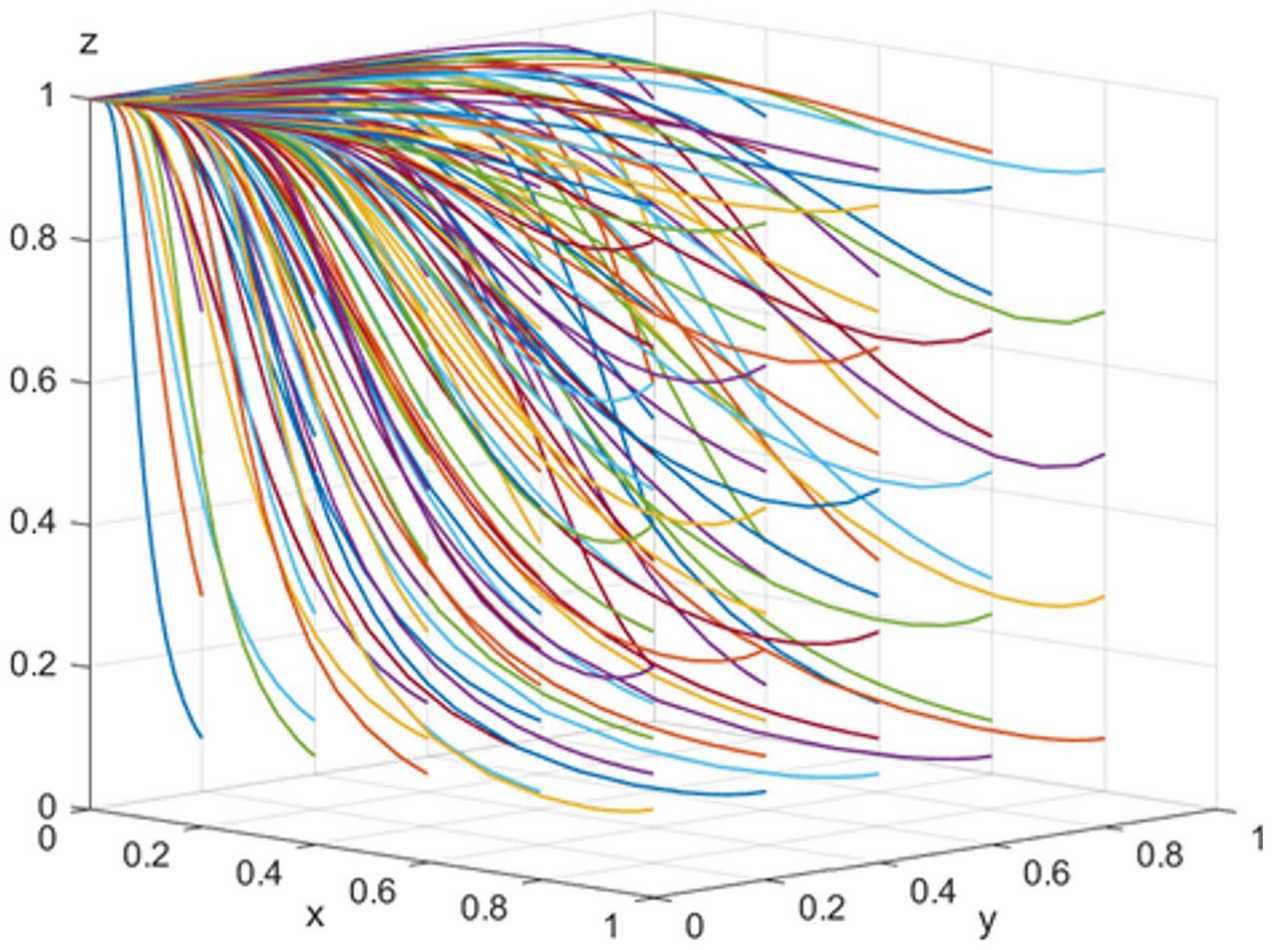
Simulation of the equilibrium point $\left(0,0,1\right)$ parameter.

*E_4_ (0,0,1)* is the evolutionarily stable equilibrium point when the conditions *C_1_ − K − V < 0, C_3_ + K − R_2_ + T < 0, and C_5_ − C_4_ + R_3_ − R_4_ + S_2_ + S_3_ − T < 0* are met. To satisfy these conditions, let us assume the following parameters: *p = 100, T = 200, K = 100, R_2_ = 450, C_3_ = 100, C_4_ = 50, S_3_ = 40, R_3_ = 40, S_2_ = 40, R4 = 120, C_5_ = 50, C_1_ = 100, V = 500, ε = 0.5, and θ = 0.5.* As Figure 2 illustrates, the likelihood of pharmaceutical companies choosing the “positive participation” strategy and hospitals opting for the “use the bid-winning drugs” strategy decreases as evolution progresses. To encourage the centralized bulk purchasing of medicines, NHSA’s probability of selecting the “high-intensity intervention” strategy increases. This trend occurs because pharmaceutical companies benefit more from “negative participation,” hospitals gain more overall benefit from selecting non-bid-winning drugs over bid-winning drugs, and “high-intensity intervention” offers greater benefits than “low-intensity intervention.” Therefore, the advantages of implementing the “high-intensity intervention” strategy are greater than those of the “low-intensity intervention” approach. Consequently, the optimal strategies for pharmaceutical companies, public hospitals, and NHSA are “negative participation,” “use of non-bid-winning drugs,” and “high-intensity intervention,” respectively.

**Figure 3:**
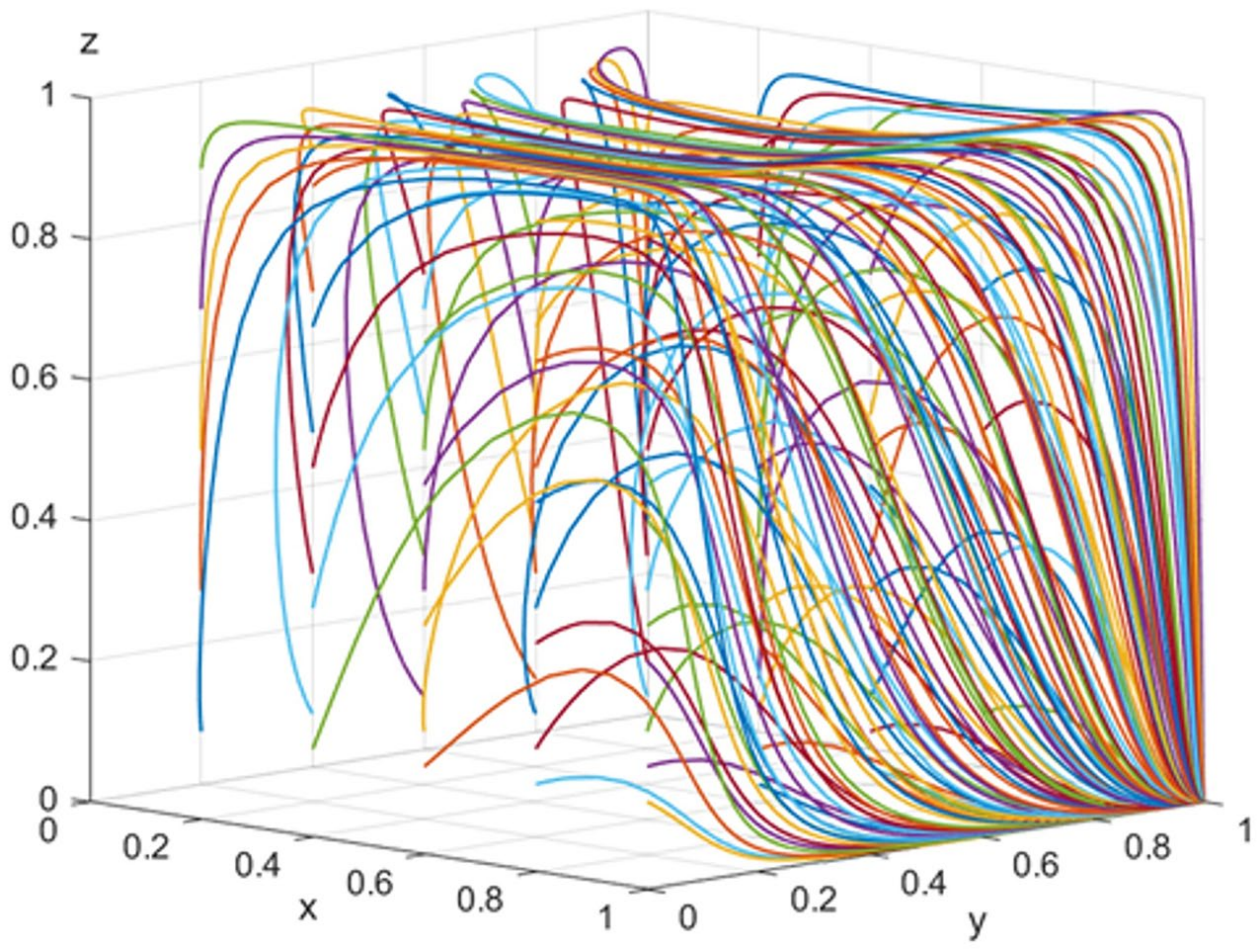
Simulation of the equilibrium point $\left(1,1,0\right)$ parameter.

*E_5_* (1,1,0) represents the evolutionary stable equilibrium point when *R_2_ − P − C_3_ − εP_1_ < 0* and *R_4_ − R_3_ − C_5_ − S_2_ + θC_4_ < 0*. To satisfy these conditions, consider the following parameters: *p = 100, T = 200, K = 100, R_2_ = 200, C_3_ = 100, C_4_ = 50, S_3_ = 40, R_3_ = 40, S_2_ = 100, R_4_ = 120, C_5_ = 50, C_1_ = 100, V = 500, ε = 0.5, and θ = 0.5*. As depicted in Figure 3, the likelihood of pharmaceutical companies adopting a strategy of “positive participation” and public hospitals opting for “using bid-winning drugs” increases as the evolution progresses. To avoid disrupting market competition, the NHSA increasingly favors a “low-intensity intervention” strategy. This trend is primarily because hospitals’ use of bid-winning drugs yields greater benefits for pharmaceutical companies, and the overall benefits for hospitals from using bid-winning drugs surpass those from using non-bid-winning drugs. Since “high-intensity intervention” elevates the NHSA’s administrative costs and adversely impacts market freedom, pharmaceutical companies, hospitals, and the NHSA should ideally adopt strategies of “positive participation,” “use of bid-winning drugs,” and “low-intensity intervention,” respectively.

**Figure 4:**
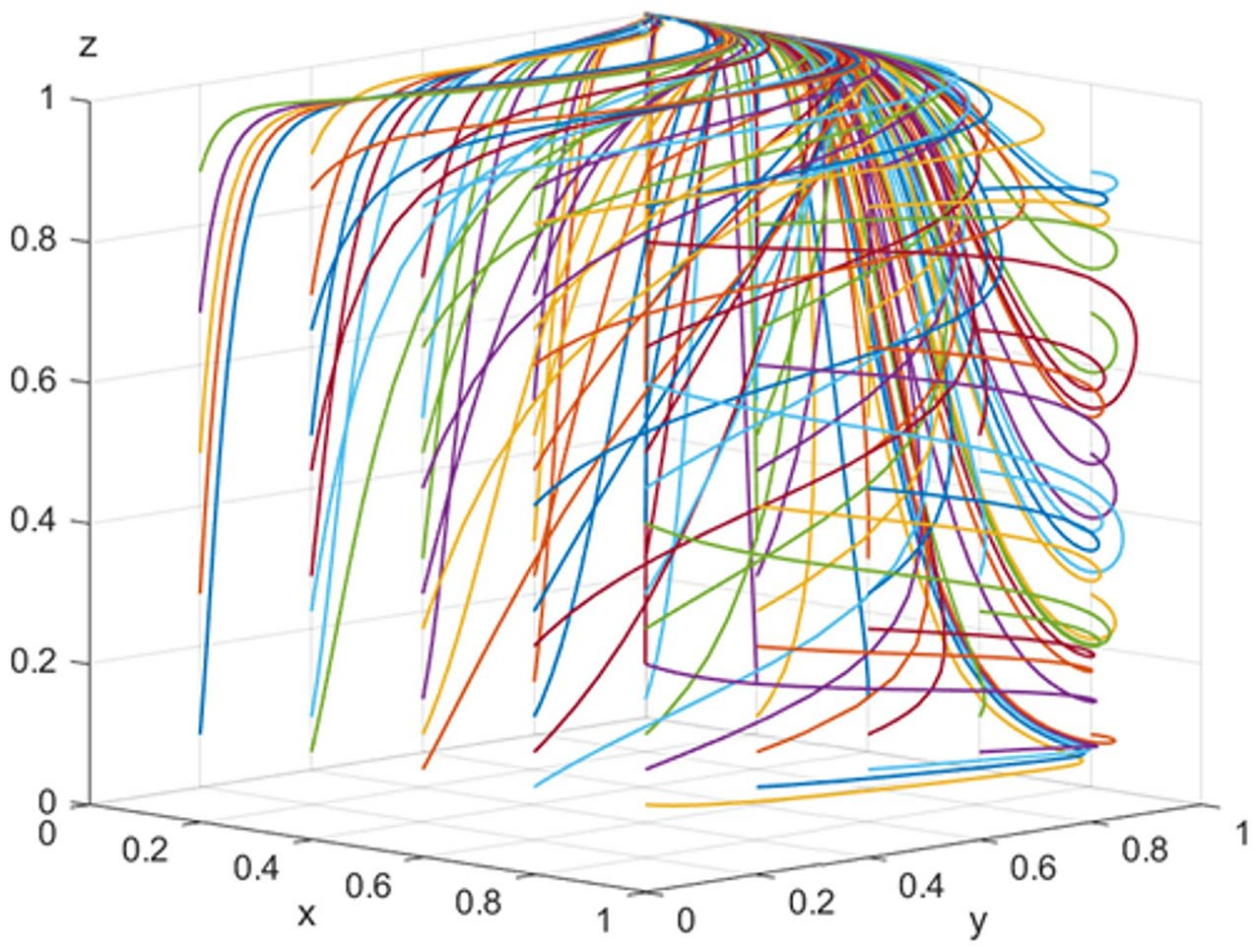
Simulation of the equilibrium point $\left(0,1,1\right)$ parameter.

*E_7_ (0,1,1)* is identified as the evolutionary stable equilibrium point when the conditions *C_1_ − V < 0, C_3_ + P_1_ − R_2_ + εP < 0, and C_4_ − C_5_ − R_3_ + R_4_ − S_2_ − S_3_ + T < 0* are met. To satisfy these conditions, consider the following parameters: *p = 100, T = 200, K = 100, R_2_ = 300, C_3_ = 100, C_4_ = 50, S_3_ = 40, R_3_ = 240, S_2_ = 100, R_4_ = 120, C_5_ = 50, C_1_ = 100, V = 500, ε = 0.5, and θ = 0.5.* As demonstrated in Figure 4, the likelihood of pharmaceutical companies adopting a “negative participation” strategy and public hospitals choosing to “use bid-winning drugs” correlates with an increase in the NHSA’s probability of opting for a “high-intensity intervention” to promote NVBP. This trend is primarily attributed to the fact that NHSA’s reputation loss outweighs its regulatory cost, and the total benefit to public hospitals from using bid-winning drugs is greater than that from using non-bid-winning drugs. However, for pharmaceutical companies, the benefit of hospitals using bid-winning drugs is less than that gained from passive participation by these companies. Consequently, the optimal strategies for pharmaceutical companies, public hospitals, and the NHSA are “negative participation,” “use of bid-winning drugs,” and “high-intensity intervention,” respectively.

### 3.2 Parametric analysis

In examining the optimization of social benefits, the optimal strategy combination emerges as (1,1,0). At this evolutionary stability point, pharmaceutical companies engage in “positive participation,” hospitals opt for “use of the bid-winning drugs,” and NHSA chooses “low-intensity intervention.” This decision is driven by several key factors. First, public hospitals, as direct beneficiaries of NVBP, can encourage pharmaceutical companies to actively participate in NVBP. This participation leads to reduced drug prices and improved accessibility by utilizing bid-winning drugs. Pharmaceutical companies involved in NVBP can attain additional economic benefits, including savings in financial expenditures and marketing costs. However, NHSA’s high-intensity intervention in the pharmaceutical industry could undermine the principle of free competition. Due to NVBP, a substantial number of small- and medium-sized generic drug manufacturers have lost market share and are facing potential insolvency. By promoting the active engagement of pharmaceutical companies in NVBP and encouraging hospitals to use bid-winning drugs, the NHSA can reduce its intervention in the pharmaceutical market, thus saving social resources and regulatory costs.

This section analyzes the sensitivity of certain parameters under the (1,1,0) decision combination: *R_2_*, the benefits of pharmaceutical companies’ negative participation in NVBP; *R_4_*, the benefits of public hospitals using non-bid-winning drugs; *C_4_*, the cost incurred by public hospitals for using bid-winning drugs; and *S_2_*, the budget allocated to public hospitals by the NHSA from the health insurance fund. When assessing the sensitivity of these parameters, the values of other parameters remain constant, as established in the equilibrium point (1,1,0) simulation discussed earlier.

#### 3.2.1 Benefits analysis of pharmaceutical companies’ negative participation in NVBP

**Figure 5:**
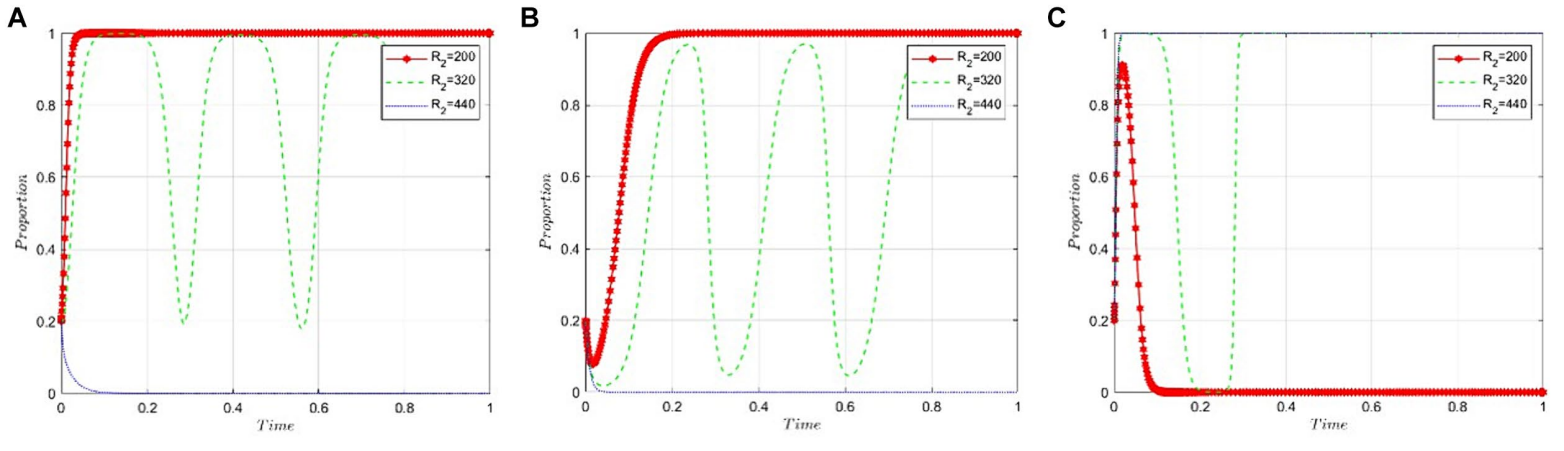
Sensitivity analysis of benefits resulting from pharmaceutical companies’ negative participation in NVBP. **(A)** Pharmaceutical companies. **(B)** Public hospitals. **(C)** NHSA.

This section delves into the sensitivity of game participants to the benefits derived from pharmaceutical companies’ negative participation in NVBP. We set the values of R_2_ at 200, 320, and 440 under the condition that *R_4_ − R_3_ − C_5_ − S_2_ + θC_4_ < 0*. Figure 5A reveals that pharmaceutical companies are inclined to adopt the “positive participation” strategy when the benefits from negative participation in NVBP are minimal. Conversely, as the benefits from negative participation incrementally increase and surpass a specific threshold, pharmaceutical companies begin to prefer the “negative participation” strategy. Figure 5B indicates that the decision-making of public hospitals is largely unaffected by the benefits of negative participation, as evidenced by the near convergence of the three curves. Figure 5C demonstrates a fluctuating trend for NHSA’s probability of adopting a “high-intensity intervention.” Initially, this probability decreases and then rises as the benefits from negative participation go beyond the threshold, eventually stabilizing with a preference for the “high-intensity intervention” strategy.

#### 3.2.2 Benefits analysis of public hospitals using non-bid-winning drugs

**Figure 6:**
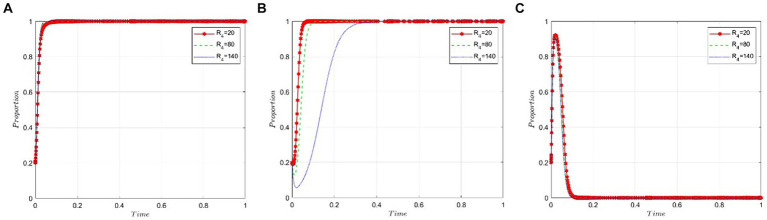
Sensitivity analysis of public hospitals’ benefits from the use of non-bid-winning drugs. **(A)** Pharmaceutical companies. **(B)** Public hospitals. **(C)** NHSA.

This section analyzes how sensitive the stakeholders in this scenario—namely, the game participants—are to the benefits public hospitals derive from using non-bid-winning drugs. The value of *R_4_* is set at 60, 120, and 180, respectively, under the condition that *R_2_ − P − C_3_ − εP* is less than zero. Figures 6A,C illustrate that the benefits public hospitals gain from using non-bid-winning drugs scarcely influence the strategic decisions of pharmaceutical companies and the NHSA. However, Figure 6B reveals a different trend: Public hospitals tend to favor bid-winning drugs when the benefits from using non-bid-winning drugs are minimal. Conversely, as the benefits of using non-bid-winning drugs increase, the likelihood of public hospitals opting for bid-winning drugs decreases steadily.

#### 3.2.3 Budget analysis of the health insurance fund allocated by the NHSA to public hospitals

**Figure 7:**
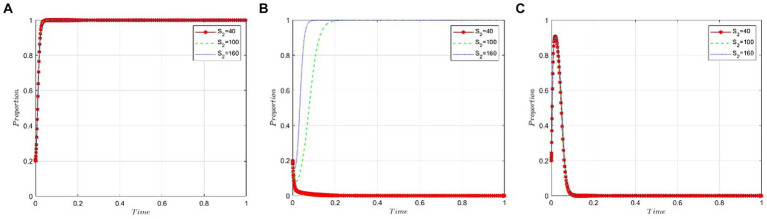
Sensitivity analysis of the budget of NHSA’s health insurance fund granted to public hospitals. **(A)** Pharmaceutical companies. **(B)** Public hospitals. **(C)** NHSA.

This section analyzes how sensitive the game participants are to the health insurance fund budget allocated to public hospitals by the NHSA. We set the value of *S_2_* at 40, 100, and 160 to satisfy the condition *R_2_ − P − C_3_ – εP < 0*. As demonstrated in Figures 7A,C, the budget allocated to public hospitals by the NHSA has little to no impact on the behavioral strategies of the pharmaceutical companies and the NHSA itself. Figure 7B indicates that as the health insurance fund budget increases, the likelihood of public hospitals opting for bid-winning drugs rises gradually.

#### 3.2.4 Cost analysis of public hospital using bid-winning drugs

**Figure 8:**
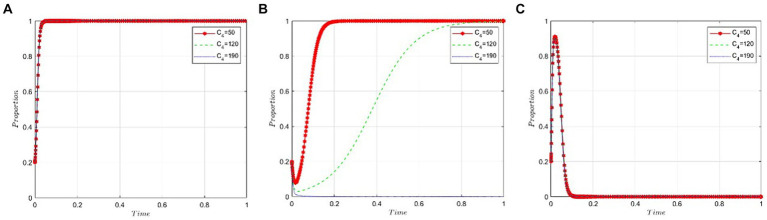
Cost analysis of public hospitals bid-winning drugs. **(A)** Pharmaceutical companies. **(B)** Public hospitals. **(C)** NHSA.

This section examines the sensitivity of game participants to the cost incurred by public hospitals when using bid-winning drugs. We set the value of *C_4_* at 50, 120, and 190 under the condition that *R_2_ − P − C_3_ – εP < 0* is satisfied. Figures 8A,C suggest that the cost to hospitals for using bid-winning drugs has a minimal impact on the behavioral strategies of both pharmaceutical companies and the NHSA. Conversely, Figure 8B shows a gradual decrease in the likelihood of hospitals choosing bid-winning drugs as their cost increases.

## 4 Discussion

This paper develops a dynamic evolutionary game model involving NHSA, public hospitals, and pharmaceutical companies. In considering the full NVBP process, the ESS (1,1,0) emerges as the most suitable. This strategy combination considers several factors: the benefits for pharmaceutical companies when they engage negatively in NVBP; the advantages for hospitals when using non-bid-winning drugs; the budget allocated to hospitals by the NHSA for health insurance funds; and the costs incurred by hospitals in using bid-winning drugs. These factors significantly influence the behavioral strategies of public hospitals. However, they exert minimal impact on the decision-making processes of pharmaceutical companies and NHSA. Specifically, the study finds the following:

1. From the standpoint of pharmaceutical companies, a significant motivating factor for active engagement in NVBP is the reduction of benefits and the escalation of costs associated with negative participation. To encourage greater involvement of these companies in NVBP, it is advisable to consider implementing legal mechanisms to regulate their negative participation. In terms of drug pricing regulations, NHSA has the authority to set drug prices covered by health insurance and to clarify the applicable mechanism for price adjustments through administrative means. Implementing this measure will effectively limit the financial gains pharmaceutical companies derive from the sale of non-bid-winning drugs and restrict their ability to generate profits through high-price tactics. Statutory price setting will establish a clear legal threshold for profits from the sale of non-bid-winning drugs, enabling companies to adapt their business strategies accordingly. The payment cycle regulations allow the NHSA to mandate payment cycles with an emphasis on extending the cycle for non-bid-winning drugs. By imposing regulatory constraints, extending the payment cycle subjects pharmaceutical companies to higher financial obligations, thus increasing the costs associated with negative participation in NVBP. This regulatory measure will encourage pharmaceutical companies to participate more positively in NVBP to avoid increased financial costs. Given the potential use of commercial bribery and other illicit methods by pharmaceutical companies to market non-bid-winning drugs, the NHSA may enhance sanctions for unethical conduct by reinforcing anti-commercial bribery regulations. This measure will not only discourage pharmaceutical companies from profiting through illicit channels but also increase the legal consequences of their involvement in NVBP. This encourages a more vigilant and proactive approach to adhering to procurement regulations. By rigorously implementing these legal strategies, pharmaceutical companies can be compelled to alter their perspectives more comprehensively and decisively, encouraging greater compliance and activity in NVBP. This will contribute to the rational operation of the health insurance funds system.
2. From the perspective of public hospitals, utilizing bid-winning drugs can lead to decreased drug prices, social benefits for NHSA, and a reduction in information asymmetry between pharmaceutical companies and public hospitals in the market. These factors collectively contribute to enhanced drug accessibility. When examining NVBP (Non-Voluntary Bid Participation) from a legal standpoint, public hospitals are bound by numerous restrictions and guidelines. To encourage hospitals to utilize bid-winning drugs more actively, the NHSA should, within legal confines, establish a policy that mandates a moderate increase in the health insurance fund for hospitals’ budgets proportional to their utilization of bid-winning drugs. This policy implementation should be in strict adherence to the law. Pharmaceutical companies should actively engage in reducing their product prices, adhering to legal constraints, and exchanging quantity for price to meet the criteria for NVBP. To optimize drug prices within the legal framework, the NHSA should negotiate with pharmaceutical companies and, through legal processes and regulations, minimize the cost for hospitals using bid-winning drugs. The NHSA can also address potential commercial bribery between pharmaceutical companies and hospitals by establishing a reporting mechanism, an incentive and penalty system, and a monitoring mechanism for the pharmaceutical market. To ensure the pharmaceutical market’s integrity and transparency, the NHSA should promptly administer statutory administrative disciplinary actions and take legal violations seriously in accordance with the rule of law. Additionally, to promote the coordinated development of the entire healthcare system, the NHSA may, within legal limits, implement an administrative reward and commendation system. This system would incentivize hospitals to use bid-winning drugs and encourage positive participation in NVBP from both drug enterprises and hospitals.
3. From the NHSA’s perspective, various lawful measures may be employed to encourage hospitals to prioritize the use of bid-winning drugs, thereby safeguarding the integrity and impartiality of the pharmaceutical industry. The NHSA should incentivize public hospitals to legally utilize bid-winning drugs. The use of these drugs by hospitals will enable NHSA to accurately determine the required quantity of drugs and facilitate centralized negotiations with pharmaceutical companies. This will ensure fair and lawful price reductions for drugs within the established legal framework. To ensure the efficient operation of the healthcare system and encourage more active participation in NVBP by pharmaceutical companies and public hospitals, the NHSA may utilize regulatory-mandated measures, including oversight and statutory subsidy policies. As the number of participating pharmaceutical companies and hospitals increases, NHSA intends to gradually decrease subsidies and oversight in accordance with the law, aiming to mitigate the operational expenses of the NVBP system. In line with the statutory framework, the NHSA is authorized to establish incentives that comply with regulations. These incentives include prepayment by the health insurance fund, volume–price linkage, and timely reimbursement as mandated by regulations. The goal is to accelerate price reductions in China’s generic drug market and foster rational competition within the pharmaceutical industry in a lawful manner. It is crucial to recognize that before the widespread occurrence of the “patent cliff” in China’s pharmaceutical industry, NHSA’s subsidy policies and regulatory measures must adhere to domestic legislation and regulations. This ensures integrity and equity while being executed in accordance with the rule of law.

## 5 Conclusion

Administrative regulation encompasses a range of actions and behaviors through which the state exercises control over economic activities. It regulates socio-economic activities and fosters the coordinated development of the national economy, using its administrative authority as the regulatory operation’s foundation. The operation of administrative regulation must adhere to various laws and regulations, including but not limited to administrative, economic, and medical laws, to ensure legality and fairness.

From the perspective of administrative regulation, the government’s use of NVBP is an effective strategy to reduce drug costs and improve drug availability. To protect public interests, the NHSA may use administrative measures to regulate drug prices legally, ensuring a balance between price and quality. Additionally, regulations should consider implementing an adaptive administrative framework to guide the quality and affordability of generic drugs, promoting fair market competition.

This paper focuses on the design of the participant interaction mechanism within NVBP, particularly the collaborative decision-making process involving multiple stakeholders. It advocates establishing a legal cooperative mechanism among pharmaceutical companies, hospitals, and the NHSA. The goal is to promote joint efforts in drug accessibility by clarifying each party’s rights and responsibilities.

Moreover, this paper highlights the need to refine research methodologies, emphasizing targeted legal measures to support NVBP. Employing an evolutionary game framework allows for a quantitative analysis of how NHSA balances economic benefits and drug prices, guiding stakeholders to assume responsibility through administrative mechanisms. This approach aids in understanding the evolutionary trajectory and stabilization strategies of multi-stakeholder interactions within the legal framework more accurately. Future research should include additional stakeholders, such as interactions between originator and generic drugs and between domestic and foreign pharmaceutical companies. It is crucial to frame the proposed actions within the rule of law to ensure the viability and effectiveness of legal provisions. Although limited in scope, this paper’s methodology and findings may provide valuable insights for other countries aiming to enhance drug accessibility and procurement.

## Data availability statement

The raw data supporting the conclusions of this article will be made available by the authors, without undue reservation.

## Author contributions

SL: Data curation, Formal analysis, Funding acquisition, Validation, Writing – review & editing. XL: Writing – original draft, Data curation, Formal analysis. ZH: Conceptualization, Methodology, Supervision, Writing – original draft, Writing – review & editing. ZZ: Software development, Funding acquisition, Validation, Writing – review & editing. ZF: Conceptualization, Writing – original draft, Funding acquisition, Resources.
